# Transfascial suture fixation technique in laparoscopic repair of inguinal hernia

**DOI:** 10.1111/ases.12715

**Published:** 2019-05-16

**Authors:** Shun‐Yan B Chan

**Affiliations:** ^1^ Department of Surgery Tseung Kwan O Hospital Hong Kong

**Keywords:** hernia, laparoscopic, mesh

## Abstract

**Introduction:**

This study presents the initial results of a transfascial suture mesh fixation technique. This method was devised to reduce operative costs and foreign body‐associated risks while embracing the benefits of fixation in laparoscopic inguinal hernia repair.

**Materials and Surgical Technique:**

Patients undergoing laparoscopic inguinal hernia repair with transfascial suture fixation (TRANSFIX) in our center between March 2017 and March 2018 were retrospectively reviewed. The procedure is orchestrated by a reusable fascial closure device sequentially puncturing the fascia vertically through the inferior port site and guiding a polypropylene thread through the mesh. The thread is retrieved from the extraperitoneal plane with the device, creating an extracorporeal suture loop to embed a surgical knot at the subcutaneous layer of the port site.

**Discussion:**

In its first year after introduction, 16 TRANSFIX were performed. All patients were men (mean age, 62.6 years). Thirteen hernias (81.3%) were first occurrence, and three (18.8%) were recurrent. Twelve (75.0%) were direct hernias, and three (18.8%) were indirect hernias; one patient presented with concurrent direct and indirect hernia. Median operating time was 41.5 minutes for unilateral repair and 73.0 minutes for bilateral. Median blood loss was 5 mL. One patient had a seroma after unilateral indirect hernia repair. After a median follow‐up of 15.5 months (range, 9‐21 months), no patient had chronic pain, wound infection, hematoma, or recurrence. Instrumental cost reduction per operation was between $150 and $300. TRANSFIX appears to be a safe and economical mesh fixation method.

## 1 INTRODUCTION

The success of laparoscopic repair has been celebrated in the field of hernia surgery over the past three decades. While its promising clinical outcomes have fueled a progressive course of technical and instrumental advancements, the controversy regarding mesh fixation remains unsettled. Studies on recurrence after laparoscopic repair have attributed it to improper fixation and mesh migration, and as a result, mesh fixation with various devices has become a widespread practice.^1^, ^2^, ^3^, ^4^ However, in recent years, encouraging early results have bolstered the concept of non‐fixation, but the long‐term benefits are unclear if mesh migration occurs; moreover, current data are insufficient to support its widespread use.^5^, ^6^

Despite being widely employed, fixation devices are not free of risks and often raise cost concerns. Reported complications associated with popular tacking staplers and tissue glue, including chronic pain, nerve entrapment, and foreign body inflammatory reactions, have prompted caution and scrutiny. Common commercial fixation devices are estimated to add a cost of $120 per procedure.^7^ The prevalence of inguinal hernia worldwide is high, entailing significant operative costs and placing substantial economic burdens on patients and health‐care systems. The transfascial suture fixation (TRANSFIX) technique discussed in this study has been devised to provide a fixation method that prevents mesh migration while minimizing operative costs and foreign body‐associated risks.

## 2 MATERIALS AND SURGICAL TECHNIQUE

**Figure 1:**
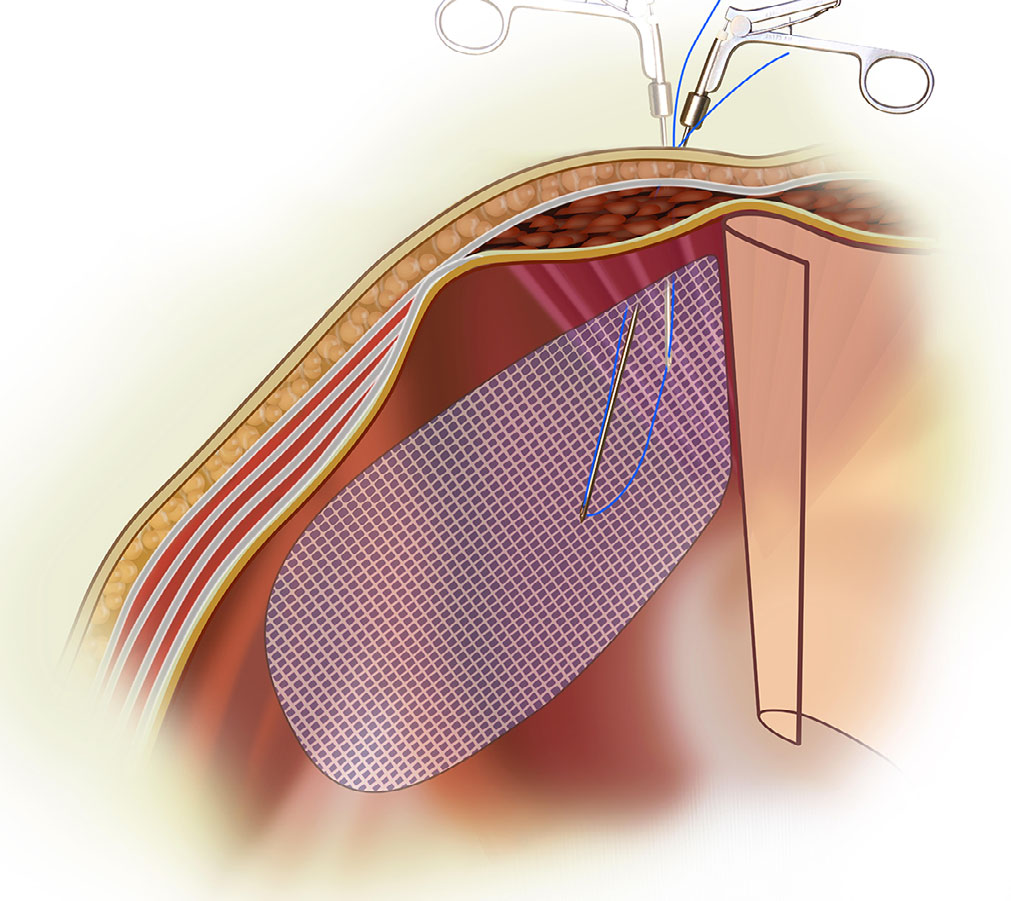
Illustration of polypropylene thread inserted into the preperitoneal space with a fascial closure device and retrieved with a second puncture to create a loop

**Figure 2:**
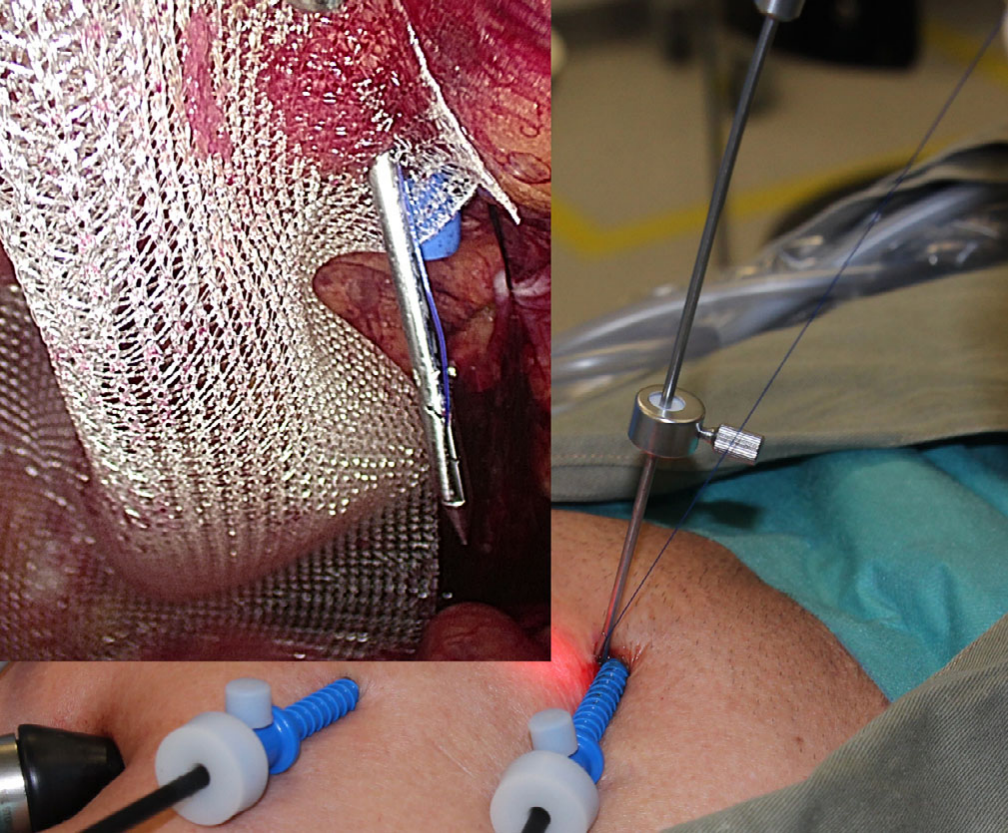
External and laparoscopic views of the first transfascial puncture through the inferior port wound while guiding the polypropylene thread into the preperitoneal space

**Figure 3:**
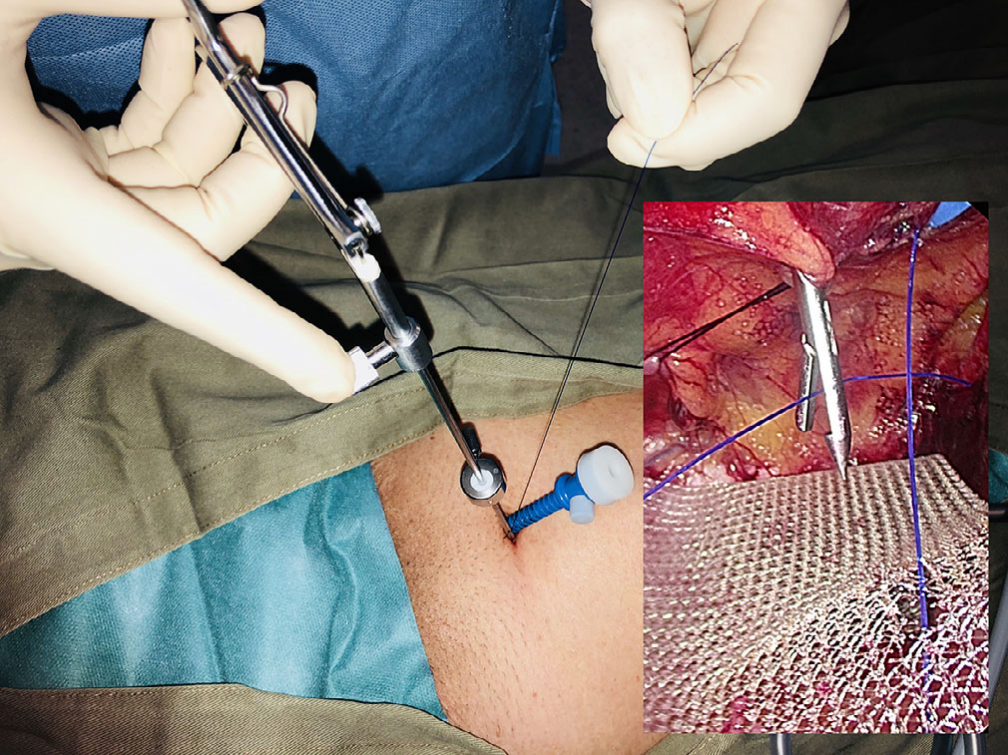
External and laparoscopic views of the second transfascial puncture while retrieving the polypropylene thread from preperitoneal space

**Figure 4:**
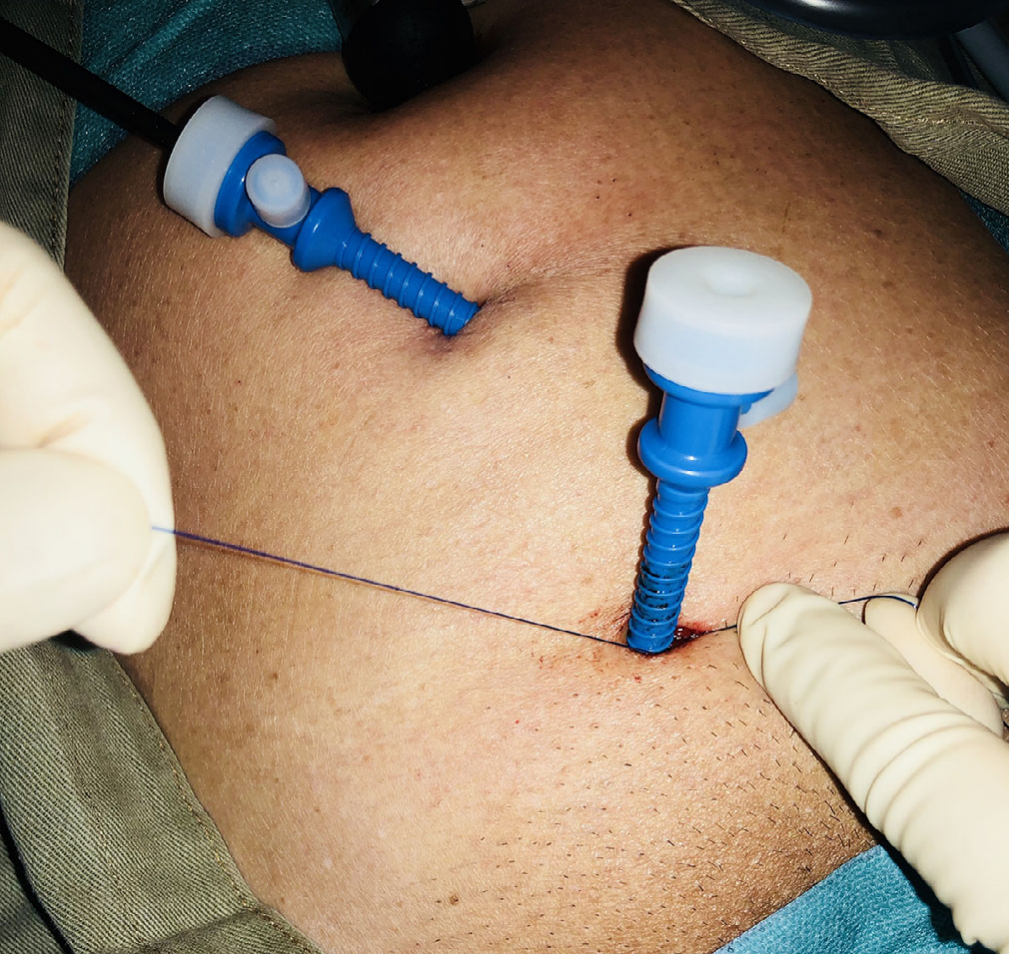
Surgical knot created and embedded in the inferior port wound to limit the need for additional stab incisions

The TRANSFIX procedure is performed with the patient under general anesthesia. Three ports are inserted along midline of the abdomen, with one 10‐mm subumbilical port, one 7‐mm port at the lower midline at the level of the anterior superior iliac spine, and one‐5 mm port midway between the other ports. The preperitoneal plane is developed and the hernia sac dissected under 8‐mm Hg preperitoneal CO_2_ pressure. After reduction of the hernial sac and parietalization, a lightweight polyvinylidene fluoride or polypropylene mesh is placed to cover the myopectineal orifice. Transfascial fixation begins with the attachment of a 15‐cm long 2‐0 polypropylene thread to the jaw of a fascial closure device (Karl Storz, Tuttlingen, Germany). The device punctures the fascia vertically through the 7‐mm inferior port wound adjacent to the 5‐mm in situ trocar (Figures 1 and 2). The tip of the device enters into the laparoscopic view of the preperitoneal space at the midline above the pubic bone and punctures the superior‐medial corner of the mesh, guiding the polypropylene thread through. The fascial closure device is then withdrawn, leaving the thread traversing the mesh. Next, the fascial closure device is reinserted via the same lower midline puncture site to retrieve the thread's loose end from the wound to form a loop that anchors the mesh to the anterior wall (Figure 3). To complete the TRANSFIX, an extracorporeal surgical knot can be secured at the subcutaneous layer in the wound (Figure 4). This procedure can be repeated at different puncturing angles to create multiple loops to prevent mesh rotation or rolling. The procedure concludes with the closure of skin wounds after exsufflation.

Patients who had undergone laparoscopic inguinal hernia repair using the TRANSFIX approach in a single center between March 2017 and March 2018 were retrospectively reviewed. Electronic and printed records of TRANSFIX procedures carried out during the 1‐year period were retrieved. All surgeries were performed by a single surgeon. Cases that employed additional fixation methods such as staples or tissue glue were excluded from this study. Patients' demographics and hernia characteristics, as well as perioperative Parameters such as operative time, blood loss, and length of hospital stay, were analyzed. Complications including wound infection, hematoma formation, recurrence, and chronic pain were evaluated up to 21 months postoperatively.

## 3 DISCUSSION

Laparoscopic inguinal hernia surgery was introduced in the early 1990s.^8^ In the early years after its introduction, hernia recurrence and mechanisms of mesh migration and folding were widely studied. Efforts to minimize mesh failure led to the development of a variety fixation techniques, especially in the 1990s and 2000s. A plethora of absorbable, nonabsorbable stapling, and non‐mechanical fixation devices were employed. However, in the past decade, controversy has emerged with regard to mesh fixation. In 2000, Tam et al conducted a meta‐analysis of fixation (staple vs none) that evaluated six randomized controlled trials with a total of 932 patients.^9^ They reported no difference in the incidence of recurrence (odds ratio = 2.01, 95% confidence interval: 0.37‐11.02) or complications (odds ratio = 0.73, 95% confidence interval: 0.51‐1.05) between the groups. They suggested that lack of fixation is associated with lower operative costs and a shorter operative time and hospital stay. Most of the studies examined by Tam et al had a median follow‐up period of less than 12 months. Several other trials similarly found that there was no significant difference between having and not having fixation in terms of short‐term to midterm recurrence.^5^, ^6^ Long‐term results are insufficient to draw further conclusions. Dehal et al challenged the idea of non‐fixation after reviewing 343 patients who had undergone bilateral total extraperitoneal repair without fixation and observing a higher recurrence rate than in the literature.^10^ Mesh fixation is currently performed in many centers worldwide, with ongoing research being carried out to evaluate the effectiveness and risks of evolving fixation devices.^11^

Suture fixation requires simple reusable instruments and minimizes foreign body introduction. Golash previously described a different suture fixation technique that required multiple stab incision wounds in the lateral spaces of the preperitoneal region and found it to be safe.^12^ In the current study, a method using embedded knots in the *in situ* inferior trocar site was developed and employed to limit the need for additional incisions and to reduce wound pain. Polypropylene sutures were selected because they are nonabsorbable and made from monofilament. Fixation points were placed at the lower midline for two main reasons. Firstly, it is a “safe zone” that is a comfortable distance from the iliac vessels inferiorly and the nerve structures laterally.^13^ Secondly, this positioning enables medial anchorage in the Hesselbach Triangle, thereby preventing the mesh from laterally migrating and resulting in the most common medial recurrence defect. Fixation at the level just above the iliopubic tract also reduces the chance of peritoneum crawling up from inferior spaces. Anchorage provides extra benefits with regard to bilateral mesh handling, as manipulation of non‐fixed mesh on one side often leads to displacement of the opposite side mesh.

Additionally, puncturing may present some risk, especially to the bladder, which can be injured if it is not adequately decompressed preoperatively by voiding or catheterization. Precautions to prevent overshooting during vertical puncture are advised to avoid breaching the peritoneum and causing visceral injury. The screws on the fascial closing forceps can be adjusted to limit the puncture depth. Also, heavyweight polypropylene mesh is generally more difficult to penetrate than lightweight mesh. To facilitate easy puncture of heavyweight mesh, a 3‐mm slit can be made with laparoscopic scissors near the medial corner of the mesh.

In total, 16 TRANSFIX procedures were performed on 10 patients in its first year after introduction. Six were bilateral repair procedures, and four were unilateral repair. All patients were men, and their mean age was 62.6 years. Thirteen repairs (81.3%) were performed on patients with first occurrence hernias, and three repairs (18.8%) were on recurrent hernias after open Lichtenstein repair (Two bilateral, one unilateral). Twelve (75.0%) were direct hernias, and three (18.8%) were indirect hernia; one patient presented with concurrent direct and indirect hernias. Median operating time was 41.5 minutes (range, 35‐72 minutes) for unilateral repair and 73.0 minutes (range, 58‐142 minutes) for bilateral repair. Median blood loss was 5 mL (range, 2‐30 mL). After an indirect hernia repair, one patient had a seroma that was managed conservatively and subsided after 6 weeks. All patients were observed overnight for assessment of pain control and potential complications; they were discharged the next day. No patients required readmission. There was no wound infection or hematoma formation. All patients had their first clinical assessment 2 to 6 weeks after discharge and had follow‐ups at the clinic every 3 or 4 month. Clinical assessments of pain, use of analgesics, wound condition, presence of complications, and recurrence were conducted at these follow‐up appointments. Bedside ultrasound was performed to diagnose seroma or hematoma if groin or scrotal swelling was detected. After a median follow‐up of 15.5 months (range, 9‐21 months), no patient complained of chronic pain, and none had required analgesics beyond 4 weeks postoperatively. No complication or recurrence was noted.

Based on these initial results, the TRANSFIX technique appears to be safe and without recurrence or chronic pain. Operative time and blood loss are comparable to procedures that do not use fixation. Because locally available commercial fixation devices were used, cost reduction per operation was between $150 and $300. Additionally, the TRANSFIX technique can theoretically be applied to transabdominal preperitoneal repair. Given the promising initial results and significant cost reduction, further efforts are encouraged to evaluate the long‐term clinical outcomes and economic benefits of TRANSFIX.

## CONFLICT OF INTERESTS

The author has no conflicts of interest and sources of funding to declare.

## References

[1] Lowham AS , Filipi CJ , Fitzgibbons RJJ , et al. Mechanisms of hernia recurrence after preperitoneal mesh repair. Traditional and laparoscopic. Ann Surg. 1997;225:422‐431. 9114802 10.1097/00000658-199704000-00012 PMC1190751

[2] Phillips EH , Rosenthal R , Fallas M , et al. Reasons for early recurrence following laparoscopic hernioplasty. Surg Endosc. 1995;9(2):140‐144; discussion 144‐145. 1995 Feb;9(2):140‐4; discussion 144‐5. 7597581 10.1007/BF00191954

[3] Taylor C , Wilson T . Long‐term results of laparoscopic totally extraperitoneal inguinal herniorrhaphy. ANZ J Surg. 2005;75(8):637‐639. 16076322 10.1111/j.1445-2197.2005.03487.x

[4] Felix E , Scott S , Craftton B , et al. Causes of recurrence after laparoscopic hernioplasty. A multicenter study. Surg Endosc. 1998;12(3):226‐231. 9502701 10.1007/s004649900640

[5] Claus CMP , Rocha GM , Campos ACL , et al. Prospective, randomized and controlled study of mesh displacement after laparoscopic inguinal repair: fixation versus no fixation of mesh. Surg Endosc. 2016;30(3). 10.1007/s00464-015-4314-7 26092029

[6] Buyukasi K , Ari A , Akce B , Tatar C , Segmen O , Bektas H . Comparison of mesh fixation and non‐fixation in laparoscopic totally extraperitoneal inguinal hernia repair. Hernia. 2017;21(4):543‐548. 28214943 10.1007/s10029-017-1590-2

[7] Ferzli GS , Frezza EE , Pecoraro AM Jr , Ahern KD . Prospective randomized study of stapled vs. unstapled mesh in laparoscopic preperitoneal inguinal hernia repair. J Am Coll Surg. 1999;188(5):461‐465. 10235572 10.1016/s1072-7515(99)00039-3

[8] Dulucq J , Wintringer P , Mahajna A . Laparoscopic totally extraperitoneal inguinal hernia repair: lessons learned from 3,100 hernia repairs over 15 years. Surg Endosc. 2009;23(3):482‐486. 18810548 10.1007/s00464-008-0118-3

[9] Tam K , Liang H , Chai C . Outcomes of staple fixation of mesh versus nonfixation in laparoscopic total extraperitoneal inguinal repair: a meta‐analysis of randomized controlled trials. World J Surg. 2010;34(12):3065‐374. 20714896 10.1007/s00268-010-0760-5

[10] Dehal A , Woodward B , Johna S , Yamanishi F . Bilateral laparoscopic totally extraperitoneal repair without mesh fixation. JSLS. 2014;18(3):e2014.00297. 10.4293/JSLS.2014.00297 PMC4154423 25392633

[11] Horisberger K , Jung MK , Zingg U , Schöb O . Influence of type of mesh fixation in endoscopic totally extraperitoneal hernia repair (TEP) on long‐term quality of life. World J Surg. 2013;37(6):1249‐1257. 23604341 10.1007/s00268-013-1974-0

[12] Golash V . Technique of suturing the mesh in laparoscopic total extra peritoneal (TEP) repair of inguinal hernia. Surgeon. 2004;2(5):264‐272. 15570845 10.1016/s1479-666x(04)80095-7

[13] Yang XF , Liu JL . Anatomy essentials for laparoscopic inguinal hernia repair. Ann Transl Med. 2016 Jun;4(19):372. 27826575 10.21037/atm.2016.09.32 PMC5075841

